# High prevalence of dyslipidemia and associated risk factors among rural Chinese adults

**DOI:** 10.1186/1476-511X-13-189

**Published:** 2014-12-12

**Authors:** Guo-Zhe Sun, Zhao Li, Liang Guo, Ying Zhou, Hong-Mei Yang, Ying-Xian Sun

**Affiliations:** Department of Cardiovascular Medicine, The First Hospital of China Medical University, Nanjing Street NO. 155, Heping District, Shenyang, 110001 Liaoning China

**Keywords:** Dyslipidemia, Prevalence, Risk factors, Rural China

## Abstract

**Background:**

Dyslipidemia is a key independent modifiable risk factor for Cardiovascular Disease, which is a leading contributor to morbidity and mortality in most developed and developing countries. This study was designed to investigate the current epidemiological features of dyslipidemia among adults in rural China.

**Methods:**

Between January 2013 and August 2013, we conducted a cross-sectional study involving 11,956 subjects with age ≥35 years in a general Chinese population. Permanent residents of the population were invited to participate in the study and the response rate was at 85.3%. Dyslipidemia was identified based on serum lipids levels following the standards proposed by the National Cholesterol Education Program Adult Treatment Panel III. Multivariate logistic regression analysis was used to evaluate the associated risk factors for dyslipidemia.

**Results:**

Within the study population, 16.4% had high TC, 13.8% had low HDL-C, 7.6% had high LDL-C, and 17.3% had high TG concentrations. Prevalence of lipid abnormality (including borderline dyslipidemia and dyslipidemia) was 47.8%, 13.8%, 25.7% and 30.7% for TC, HDL-C, LDL-C and TG, respectively. Detailed analysis indicated that 36.9% of this population had at least one type of dyslipidemia and 64.4% had at least one type of abnormal lipid concentration. Thus, this study observed an alarmingly higher prevalence of lipid abnormality, in a relatively large population, compared to previous studies. Further, we determined that not all of the risk factors studied, including age, gender, hypertension, diabetes mellitus, obesity, smoking, drinking, education level, marital status, and family income, influenced dyslipidemia to the same extent.

**Conclusions:**

Our present study, in a population of 11,956 adults in Liaoning Providence, demonstrated a very high prevalence of dyslipidemia, which represented an alarming rise since the publication of our previous study and other similar studies around the world, which report lower levels. We also examined various risk factors for dyslipidemia, many of which are modifiable risk factors for Cardiovascular Disease (CVD), to provide a comprehensive view that will help in designing strategies to slow the rapid spread and promote effective measures to treat dyslipidemia. Our ultimate goal is to prevent the increasing prevalence of lipid abnormality and reduce the burden of CVD in rural China.

## Introduction

Cardiovascular Disease (CVD) is a major cause of morbidity and a leading contributor to mortality in both developed and developing countries [1]. With rapid socioeconomic development, CVD has reached epidemic proportions in developing countries in recent decades [2–4]. China is a developing country, and has a high level of CVD mortality, accounting for nearly 40 percent of all deaths. Furthermore, CVD morbidity and mortality in China has been projected to increase substantially both in absolute numbers and as a proportion of total disease burden in the population over the next 20 years [5].

Dyslipidemia is an important modifiable risk factor for the development of atherosclerosis and CVD [6–8]. Effective management of dyslipidemia, by pharmacological treatment or lifestyle changes, is known to reduce the rate of CVD morbidity and mortality [9–11]. In the United States, the National Health and Nutrition Examination Survey (NHANES) 2003–2006 showed that 52.9% of adults had lipid abnormalities (including borderline dyslipidemia and dyslipidemia) [12]. In China, the prevalence of dyslipidemia in adults aged 18 and older was 18.6% according to the Chinese National Nutrition and Health Survey in 2002 [13]. The results from the International Collaborative Study of Cardiovascular Disease in Asia (InterAsia), conducted during 2000 to 2001, demonstrated that the prevalence in Chinese adults was 53.6% (aged 35-74 years) when borderline dyslipidemia and dyslipidemia numbers are combined [14]. With rapid economic growth and associated lifestyle changes, dyslipidemia in China has increased significantly during the past decade [15–17].

Paradoxically, surveys to monitor and measure dyslipidemia burden in a Chinese population has not been conducted in recent years and the available data on the prevalence, types, and associated factors of dyslipidemia in the general population is relatively insufficient and outdated. Therefore, the objective of this study was to obtain current data on dyslipidemia.

## Materials and methods

### Study population

Liaoning Province is located in northeast China. Between January 2013 to August 2013, a representative sample aged ≥35 years was selected to study the prevalence and natural history of cardiovascular risk factors in the countryside of Liaoning Province. The study adopted a multi-stage, stratified, randomly cluster-sampling scheme. In the first stage, 3 counties (Dawa, Zhangwu, and Liaoyang) were selected from the eastern, southern, and northern region of Liaoning province. In the second stage, one town near the city was randomly selected from each county (a total of 3 towns). In the third stage, 6-8 villages from each town were randomly selected (a total of 26 rural villages). Pregnant participants, those with malignant tumors, and those with mental disorders were excluded. All eligible permanent residents aged ≥35 years from each village were invited to attend the study (a total of 14016 participants). Of those, 11956 participants agreed and completed the study giving a response rate of 85.3%. The study was approved by the Ethics Committee of China Medical University (Shenyang, China). All procedures were performed in accordance with ethical standards. Written consent was obtained from all participants after they had been informed of the objectives, benefits, medical items, and confidentiality agreement of personal information. If the participants were illiterate, we obtained the written informed consents from their proxies.

### Data collection and measurements

Before the survey began, all investigators attended organized training sessions. The training contents included the purpose of this study, how to properly administer the questionnaire, the standard method of measurement, the importance of standardization, and the study procedures. A strict test was administered to evaluate the effectiveness of the training, and only those who scored correct on all test questions could become eligible investigators. During data collection, our inspectors received further instructions and support.

Data was collected during a single clinic visit with cardiologists and trained nurses using a standard questionnaire in face-to-face interviews. Data on demographic characteristics, medical history of hypertension and diabetes mellitus, lifestyle risk factors, educational level, marital status, and family income were obtained by interview with a standardized questionnaire. Organizationally, we adapted a central steering committee with a subcommittee for quality control.

According to American Heart Association protocol, blood pressure (BP) was measured three times at 2-min intervals after at least 5 min of rest using a standardized automatic electronic sphygmomanometer (HEM-907; Omron). The calibration of the Omron device was checked prior to use, using a standard mercury sphygmomanometer every month by two doctors according to the British Hypertension Society protocol [18]. The participants were advised to avoid caffeinated beverages and exercise for at least 30 min prior to the measurement. During the measurement, the participants were seated with the arm supported at heart level. The mean of three BP measures was calculated and used in all analyses.

Weight and height were measured to the nearest 0.1 kg and 0.1 cm, respectively, with the participants in light-weight clothing and without shoes. Waist circumference was measured at the umbilicus to the nearest 0.1 cm [19], with the participants standing and at the end of normal expiration. The body mass index (BMI) was calculated as weight in kilograms divided by the square of the height in meters.

Fasting blood samples were collected in the morning after at least 8h of fasting for all participants. Blood samples were obtained from an antecubital vein into Vacutainer tubes containing EDTA. Serum was subsequently isolated from the blood, and all serum samples were frozen at –20°C for testing at a central, certified laboratory. Fasting plasma glucose (FPG), total cholesterol, low-density lipoprotein cholesterol (LDL-C), high-density lipoprotein cholesterol (HDL-C), triglycerides, and other routine blood biochemical indexes were analyzed enzymatically on an Olympus AU640 autoanalyzer (Olym-pus, Kobe, Japan). All analytes were measured in an autoanalyzer (Bayer RA-XT, Tarrytown, NY, USA) using kits by the same company (Bayer Diagnostics, Tarrytown, NY, USA).

### Definitions

According to JNC-7 report [20, 21], hypertension is defined as SBP ≥ 140 mm Hg and/or DBP ≥ 90 mm Hg and/or use of antihypertensive medications. BMI’s is categorized into 3 groups as normal (BMI <25 kg/m^2^), overweight (25 ≤ BMI <30 kg/m^2^) and obese (BMI ≥30 kg/m^2^), according to the World Health Organization’s (WHO) criteria [22]. Abdominal obesity was defined as WC ≥ 88 cm for females and WC ≥ 102 cm for males [23]. Diabetes mellitus was diagnosed according to the WHO criteria [24]: FPG ≥ 7.0 mmol/L (126 mg/dL) and/or being on treatment for diabetes.

Dyslipidemia was defined according to the Third Report of the National Cholesterol Education Program Adult Treatment Panel III (NCEP ATP III) [24]. Borderline high and high TC was defined as having TC levels of 5.18-6.21 mmol/L (200-239 mg/dL) and ≥6.22 mmol/L (≥240 mg/dL), respectively. Low HDL-C was defined as having HDL-C levels <1.04 mmol/L (<40 mg/dL). Borderline high, high, and very high LDL-C was defined as having LDL-C levels of 3.37-4.13mmol/L (130-159 mg/dL), 4.14-4.91 mmol/L (160-189 mg/dL), and ≥4.92 mmol/L (≥190 mg/dL), respectively. Borderline high, high, and very high TG was defined as having triglyceride levels of 1.70-2.25 mmol/L (150-199 mg/dL), 2.26-5.64 mmol/L (200-499 mg/dL), and ≥5.65 mmol/L (≥500 mg/dL), respectively. Dyslipidemia was defined as having at least one of the following: high TC, high LDL-C, low HDL-C and high TG.

### Statistical analysis

Continuous variables were expressed as mean ± standard deviation (SD) while categorical data was presented as frequencies or percentages. Differences between groups were compared using Student’s *t*-test (two-tailed) or *χ*^2^ test for continuous variables and categorical variables, respectively. The percentage estimates of abnormal TC, HDL-C, LDL-C, and TG categories were calculated by age and gender-specific groups. *χ*^2^ test for trends was used to analyze the significance of an increase or decrease in prevalence across age groups. The detailed prevalence of dyslipidemia by selected characteristics was also calculated. To evaluate the association between blood lipids and risk factors, multivariate logistic regression analysis was used with abnormal serum levels of TC, HDL-C, LDL-C and TG as dependent variables and with demographics, anthropometrics, and lifestyles as the independent variables. The adjusted odds ratio (OR) was presented together with a 95% confidence interval (95% CI). All the statistical analyses were conducted with SPSS 17.0 statistical software package (SPSS, Inc., Chicago, IL, USA), and *P* values <0.05 were considered to be statistically significant.

## Results

### Characteristics of the study population

Characteristics of the study participants enrolled in this study, as stratified by gender, are shown in Table 1. Among the 11956 participants, 377 were excluded during analysis for incomplete data. A total of 11579 participants (5361 men and 6218 women) aged 35 years and older were included in this study. Mean age of the subjects was 54.4 and 53.4 years for male and female, respectively. Men had significantly lower mean values for TC and LDL-C compared with women, however, the mean concentration for HDL-C and TG showed no difference between men and women. The mean level of SBP, DBP and FBG were all significantly higher for man than women. Also, smoking, drinking status and educational level were significantly higher for men than women. About 50% of the subjects had primary school education or below and 95% of them had Han nationality. Marital statuses and family income grouped by gender are also presented.

**Table 1:** Basic characteristics of the study population*

Variables	Male (n = 5361)	Female (n = 6218)	p values
Age (years)	54.4 ± 10.8	53.4 ± 10.3	<0.001
Height (cm)	166.4 ± 6.3	155.6 ± 6.1	<0.001
Weight (kg)	68.6 ± 11.1	60.3 ± 10.1	<0.001
Waist circumstance (cm)	83.8 ± 9.8	81.3 ± 9.7	<0.001
BMI (kg/m2)	24.7 ± 3.5	24.9 ± 3.8	0.065
TC (mmol/L)	5.17 ± 1.04	5.29 ± 1.12	<0.001
HDL-C (mmol/L)	1.41 ± 0.42	1.41 ± 0.34	0.660
LDL-C (mmol/L)	2.88 ± 0.79	2.97 ± 0.84	<0.001
TG (mmol/L)	1.66 ± 1.66	1.61 ± 1.33	0.116
SBP (mmHg)	143.7 ± 22.6	140.2 ± 24.0	<0.001
DBP (mmHg)	83.8 ± 11.8	80.6 ± 11.5	<0.001
FBG (mmol/L)	5.95 ± 1.67	5.86 ± 1.60	0.004
Han nationality (%)	5075 (94.7%)	5899 (94.9%)	0.622
Current smoking state (%)			<0.001
No	2302 (42.9%)	5188 (83.4%)	
Yes	3059 (57.1%)	1030 (16.6%)	
Current drinking state (%)			<0.001
No	2926 (54.6%)	6035 (97.1%)	
Yes	2435 (45.4%)	183 (2.9%)	
Educational level			<0.001
Illiterate (1)	247 (4.6%)	752 (12.1%)	
Primary school (2)	1987 (37.1%)	2781 (44.7%)	
Middle school (3)	2514 (46.9%)	2205 (35.5%)	
High school or above (4)	613 (11.4%)	480 (7.7%)	
Marital status			0.022
Married	4943 (92.3%)	5661 (91.1%)	
Unmarried or widowed	415 (7.7%)	555 (8.9%)	
Family income (yuan/year)			0.009
<=5000	721 (13.4%)	721 (11.6%)	
5000-20000	2872 (53.6%)	3438 (55.3%)	
>20000	1768 (33.0%)	2059 (33.1%)	

*Data are expressed as means ± SD or n (%).

BMI: body mass index; TC: total cholesterol; HDL-C: high-density lipoprotein cholesterol; LDL-C: low-density lipoprotein cholesterol; TG: triglyceride; SBP: systolic blood preesure; DBP: dystolic blood preesure; FBG: fasting blood glucose.

### Prevalence of dyslipidemia

Table 2 shows the prevalence rates of abnormal lipid levels by age and gender group. Of 11579 subjects, 16.4% had high TC, 13.8% had low HDL-C, 2.0% had high LDL-C, and 1.8% had high TG concentrations. And prevalence of lipid abnormality (including borderline dyslipidemia and dyslipidemia) was 16.4%, 13.8%, 2.0% and 1.8% for TC, HDL-C, LDL-C and TG, respectively. Of these measures, 36.9% of this population had at least one type of dyslipidemia and 64.4% had at least one type of abnormal lipid concentration. Prevalence of borderline high and high TC was 31.4% and 16.4%, respectively, and the prevalence for both was significantly higher in women than in men (*P* < 0.001). The age-specific prevalence of borderline high and high TC for both men and women increased with age (*P* < 0.05). Prevalence of low HDL-C was 13.8%. The prevalence was significantly higher in men than in women (*P* < 0.001), and the age-specific prevalence decreased with age only in men (*P* = 0.008). The prevalence of borderline high, high, and very high LDL-C was 18.1%, 5.6% and 2.0%, respectively. The prevalence of borderline high, high, and very high LDL-C was significantly higher in women than in men (*P* < 0.001) and the age-specific prevalence increased with age in the group of women (*P* < 0.001). The prevalence of borderline high, high, and very high TG was 13.4%, 15.5% and 1.8%, respectively. Differences between men and women were not significant. The age-specific prevalence increased with age in women (*P* < 0.001); however, it decreased in men (*p* < 0.001). Table 3 shows the age- and gender-specific prevalence rates of high TC, low HDL-C, high LDL-C and high TG. Women were more likely than men to have high TC (18.2% vs. 14.3%, *P* < 0.001), high LDL-C (8.8% vs. 6.2%, *P* < 0.001) and less likely to have low HDL-C (10.8% vs. 17.3%, *P* < 0.001). The prevalence of high TC, LDL-C and TG increased significantly with advancing age, with the highest prevalence being observed in the group of 55–64-year-olds (21.9%, 11.1% and 19.4%, respectively), and then declined thereafter. It was worth noting that the prevalence of high TC, LDL-C and TG changed with parallelism. By contrast, the prevalence of low HDL-C declined along with advancing age, with the lowest prevalence being observed in the 55–64-year-old group (12.7%), and then increasing thereafter.

**Table 2:** **Prevalence of dyslipidemia in all subjects by age and gender group**

	TC mmol/L (mg/dL)		HDL-C mmol/L (mg/dL)	LDL-C mmol/L (mg/dL)			TG mmol/L (mg/dL)		
	5.18-6.21	≥6.22	<1.04	3.37-4.13	4.14-4.91	≥4.92	1.70-2.25	2.26-5.64	≥5.65
	(200-239)	(≥240)	(<40)	(130-159)	(160-189)	(≥190)	(150-199)	(200-499)	(≥500)
All (n = 11579)									
Total	3638 (31.4%)	1896 (16.4%)	1599 (13.8%)	2091 (18.1%)	653 (5.6%)	230 (2.0%)	1547 (13.4%)	1795 (15.5%)	212 (1.8%)
35-44	687 (25.0%)	226 (8.2%)	415 (15.1%)	331 (12.0%)	72 (2.6%)	23 (0.8%)	269 (9.8%)	366 (13.3%)	48 (1.7%)
45-54	1121 (31.1%)	554 (15.4%)	506 (14.1%)	666 (18.5%)	169 (4.7%)	56 (1.6%)	484 (13.4%)	550 (15.3%)	74 (2.1%)
55-64	1195 (34.3%)	762 (21.9%)	442 (12.7%)	725 (20.8%)	281 (8.1%)	104 (3.0%)	534 (15.3%)	609 (17.5%)	66 (1.9%)
≥65	635 (36.4%)	354 (20.3%)	236 (13.5%)	369 (21.2%)	131 (7.5%)	47 (2.7%)	260 (14.9%)	270 (15.5%)	24 (1.4%)
*p* for trend	<0.001		0.025	<0.001			<0.001		
Male (n = 5361)									
Total	1659 (30.9%)	764 (14.3%)	925 (17.3%)	898 (16.8%)	246 (4.6%)	88 (1.6%)	690 (12.9%)	818 (15.3%)	121 (2.3%)
35-44	346 (28.4%)	147 (12.1%)	242 (19.9%)	188 (15.4%)	44 (3.6%)	17 (1.4%)	155 (12.7%)	235 (19.3%)	40 (3.3%)
45-54	503 (31.0%)	250 (15.4%)	288 (17.8%)	300 (18.5%)	78 (4.8%)	19 (1.2%)	198 (12.2%)	282 (17.4%)	41 (2.5%)
55-64	526 (32.2%)	252 (15.4%)	245 (15.0%)	267 (16.3%)	81 (5.0%)	39 (2.4%)	234 (14.3%)	215 (13.1%)	32 (2.0%)
≥65	284 (32.1%)	115 (13.0%)	150 (16.9%)	143 (16.2%)	43 (4.9%)	13 (1.5%)	103 (11.6%)	86 (9.7%)	8 (0.9%)
*p* for trend	0.028		0.008	0.089			<0.001		
Female (n = 6218)									
Total	1979 (31.8%)	1132 (18.2%)	674 (10.8%)	1193 (19.2%)	407 (6.5%)	142 (2.3%)	857 (13.8%)	977 (15.7%)	91 (1.5%)
35-44	341 (22.2%)	79 (5.2%)	173 (11.3%)	143 (9.3%)	28 (1.8%)	6 (0.4%)	114 (7.4%)	131 (8.5%)	8 (0.5%)
45-54	618 (31.2%)	304 (15.4%)	218 (11.0%)	366 (18.5%)	91 (4.6%)	37 (1.9%)	286 (14.5%)	268 (13.5%)	33 (1.7%)
55-64	669 (36.2%)	510 (27.6%)	197 (10.7%)	458 (24.8%)	200 (10.8%)	65 (3.5%)	300 (16.2%)	394 (21.3%)	34 (1.8%)
≥65	351 (40.9%)	239 (27.8%)	86 (10.0%)	226 (26.3%)	88 (10.2%)	34 (4.0%)	157 (18.3%)	184 (21.4%)	16 (1.9%)
*p* for trend	<0.001		0.318	<0.001			<0.001		

Data are n (%).

TC: total cholesterol; HDL-C: high-density lipoprotein cholesterol; LDL-C: low-density lipoprotein cholesterol; TG: triglyceride.

**Table 3:** **Prevalence of dyslipidemia by selected characteristics and adjusted odds ratio (95% CI) of dyslipidemia prevalence**

Variables	High TC (≥240 mg/dl)			Low HDL-C (<40 mg/dl)			High LDL-C (≥160 mg/dl)		
	n (%)	Adjusted OR (95% CI)	p values	n (%)	Adjusted OR (95% CI)	p values	n (%)	adjusted OR (95% CI)	p values
	1896 (16.4%)			1599 (13.8%)			883 (7.6%)		
Gender									
Male	764 (14.3%)	1		925 (17.3%)	1		334 (6.2%)	1	
Female	1132 (18.2%)	1.642 (1.431, 1.884)	<0.001	674 (10.8%)	0.397 (0.347, 0.455)	<0.001	549 (8.8%)	1.625 (1.344, 1.964)	<0.001
Age group									
35-44	226 (8.2%)	1		415 (15.1%)	1		95 (3.5%)	1	
45-54	554 (15.4%)	1.849 (1.564, 2.186)	<0.001	506 (14.1%)	0.894 (0.772, 1.036)	0.137	225 (6.3%)	1.646 (1.282, 2.113)	<0.001
55-64	762 (21.9%)	2.643 (2.224, 3.142)	<0.001	442 (12.7%)	0.767 (0.651, 0.903)	0.001	385 (11.1%)	2.884 (2.246, 3.702)	<0.001
≥65	354 (20.3%)	2.355 (1.914, 2.897)	<0.001	236 (13.5%)	0.811 (0.660, 0.997)	0.047	178 (10.2%)	2.582 (1.926, 3.463)	<0.001
BMI (kg/m2)									
Normal, <25.0	879 (13.8%)	1		615 (9.7%)	1		337 (5.3%)	1	
Overweight, 25.0-29.9	824 (19.1%)	1.357 (1.210, 1.521)	<0.001	767 (17.8%)	2.085 (1.846, 2.356)	<0.001	431 (10.0%)	1.893 (1.613, 2.221)	<0.001
Obese, ≥30.0	193 (21.5%)	1.421 (1.143, 1.767)	0.002	217 (24.1%)	3.324 (2.673, 4.134)	<0.001	115 (12.8%)	2.477 (1.872, 3.278)	<0.001
Waist circumstance (cm)									
<102 (male)/88 (female)	1515 (15.2%)	1		1339 (13.4%)	1		683 (6.8%)	1	
≥102 (male)/88 (female)	381 (23.8%)	1.130 (0.958, 1.333)	0.148	260 (16.3%)	0.921 (0.762, 1.113)	0.394	200 (12.5%)	0.937 (0.754, 1.165)	0.557
Hypertension									
No	670 (11.8%)	1		755 (13.3%)	1		268 (4.7%)	1	
Yes	1226 (20.7%)	1.431 (1.279, 1.600)	<0.001	844 (14.3%)	0.928 (0.823, 1.046)	0.222	615 (10.4%)	1.583 (1.348, 1.859)	<0.001
T2DM									
No	1570 (15.1%)	1		1364 (13.1%)	1		719 (6.9%)	1	
Yes	326 (27.2%)	1.570 (1.359, 1.814)	<0.001	235 (19.6%)	1.509 (1.282, 1.777)	<0.001	164 (13.7%)	1.465 (1.213, 1.770)	<0.001
Current smoking state (%)									
No	1245 (16.6%)	1		977 (13.0%)	1		596 (8.0%)	1	
Yes	651 (15.9%)	1.051 (0.932, 1.186)	0.415	622 (15.2%)	1.242 (1.096, 1.408)	0.001	287 (7.0%)	1.106 (0.936, 1.306)	0.239
Current drinking state (%)									
No	1421 (15.9%)	1		1360 (15.2%)	1		702 (7.8%)	1	
Yes	475 (18.1%)	1.691 (1.461, 1.957)	<0.001	239 (9.1%)	0.310 (0.264, 0.365)	<0.001	181 (6.9%)	1.193 (0.968, 1.471)	0.098
Educational level									
Illiterate	249 (24.9%)	1		129 (12.9%)	1		129 (12.9%)	1	
Primary school	879 (18.4%)	0.827 (0.699, 0.978)	0.027	645 (13.5%)	0.908 (0.734, 1.122)	0.370	402 (8.4%)	0.778 (0.625, 0.970)	0.025
Middle school	606 (12.8%)	0.695 (0.577, 0.838)	<0.001	656 (13.9%)	0.813 (0.649, 1.017)	0.070	275 (5.8%)	0.735 (0.575, 0.941)	0.015
High school or above	162 (14.8%)	0.742 (0.584, 0.942)	0.014	169 (15.5%)	0.887 (0.677, 1.162)	0.386	77 (7.0%)	0.831 (0.603, 1.145)	0.257
Marital status									
Married	1682 (15.9%)	1		1475 (13.9%)	1		782 (7.4%)	1	
unmarried or widowed	212 (21.9%)	1.244 (1.043, 1.484)	0.015	124 (12.8%)	1.006 (0.813, 1.246)	0.953	101 (10.4%)	1.149 (0.906, 1.457)	0.253
Family income (yuan/year)									
<=5000	229 (15.9%)	1		177 (12.3%)	1		120 (8.3%)	1	
5000-20000	1012 (16.0%)	1.202 (1.019, 1.419)	0.029	845 (13.4%)	1.106 (0.920, 1.329)	0.284	478 (7.6%)	1.067 (0.856, 1.329)	0.564
>20000	655 (17.1%)	1.553 (1.299, 1.857)	<0.001	577 (15.1%)	1.270 (1.043, 1.547)	0.017	285 (7.4%)	1.249 (0.983, 1.588)	0.069
**Variables**	**High TG (≥200 mg/dl)**			**Dyslipidemia (any one kind of dyslipidemia)**					
	**n (%)**	**Adjusted OR (95% CI)**	**p values**	**n (%)**				**Adjusted OR (95% CI)**	**p values**
	2007 (17.3%)			4267 (36.9%)					
Gender									
Male	939 (17.5%)	1		2038 (38.0%)				1	
Female	1068 (17.2%)	0.955 (0.836, 1.092)	0.500	2229 (35.8%)				0.847 (0.765, 0.937)	0.001
Age group									
35-44	414 (15.0%)	1		830 (30.2%)				1	
45-54	624 (17.3%)	0.986 (0.854, 1.139)	0.847	1299 (36.1%)				1.183 (1.058, 1.323)	0.003
55-64	675 (19.4%)	1.026 (0.879, 1.198)	0.742	1442 (41.4%)				1.386 (1.229, 1.563)	<0.001
≥65	294 (16.9%)	0.866 (0.711, 1.054)	0.151	696 (39.9%)				1.278 (1.099, 1.487)	0.001
BMI (kg/m2)									
Normal, <25.0	618 (9.7%)	1		1780 (27.9%)				1	
overweight, 25.0-29.9	1086 (25.2%)	2.646 (2.358, 2.970)	<0.001	1983 (46.0%)				2.043 (1.872, 2.230)	<0.001
Obese, ≥30.0	303 (33.7%)	3.080 (2.523, 3.761)	<0.001	504 (56.1%)				2.814 (2.367, 3.347)	<0.001
Waist circumstance (cm)									
<102 (male)/88 (female)	1497 (15.0%)	1		3452 (34.6%)				1	
≥102 (male)/88 (female)	510 (31.9%)	1.473 (1.260, 1.723)	<0.001	815 (51.0%)				1.142 (0.997, 1.309)	0.056
Hypertension									
No	675 (11.9%)	1		1709 (30.2%)				1	
Yes	1332 (22.5%)	1.595 (1.425, 1.785)	<0.001	2558 (43.2%)				1.321 (1.213, 1.439)	<0.001
T2DM									
No	1556 (15.0%)	1		3572 (34.4%)				1	
Yes	451 (37.6%)	2.634 (2.298, 3.020)	<0.001	695 (57.9%)				2.053 (1.808, 2.331)	<0.001
Current smoking state (%)									
No	1266 (16.9%)	1		2703 (36.1%)				1	
Yes	741 (18.1%)	1.309 (1.162, 1.475)	<0.001	1564 (38.2%)				1.238 (1.129, 1.358)	<0.001
Current drinking state (%)									
No	1536 (17.1%)	1		3328 (37.1%)				1	
Yes	471 (18.0%)	1.002 (0.869, 1.154)	0.983	939 (35.9%)				0.826 (0.739, 0.922)	0.001
Educational level									
Illiterate	173 (17.3%)	1		423 (42.3%)				1	
Primary school	882 (18.5%)	1.130 (0.933, 1.370)	0.212	1857 (38.9%)				0.916 (0.791, 1.060)	0.239
Middle school	756 (16.0%)	1.016 (0.827, 1.248)	0.879	1583 (33.5%)				0.789 (0.675, 0.924)	0.003
High school or above	196 (17.9%)	1.101 (0.856, 1.416)	0.453	404 (37.0%)				0.853 (0.703, 1.035)	0.108
Marital status									
Married	1850 (17.4%)	1		3873 (36.5%)				1	
Unmarried or widowed	156 (16.1%)	0.908 (0.746, 1.105)	0.336	391 (40.3%)				1.128 (0.973, 1.307)	0.110
Family income (yuan/year)									
<=5000	259 (18.0%)	1		516 (35.8%)				1	
5000-20000	1072 (17.0%)	0.894 (0.760, 1.053)	0.179	2289 (36.3%)				1.114 (0.980, 1.267)	0.098
>20000	676 (17.7%)	0.988 (0.828, 1.178)	0.890	1462 (38.2%)				1.339 (1.165, 1.539)	<0.001

BMI: Body mass index; FBG: fasting blood glucose; IFG: impaired fasting blood glucose; T2DM: type 2 diabetes mellitus; TC: total cholesterol; HDL-C: high-density lipoprotein cholesterol; LDL-C: low-density lipoprotein cholesterol; TG: triglyceride.

### Factors associated with dyslipidemia

Table 3 shows the prevalence of high TC, low HDL-C, high LDL-C and high TG according to different characteristics of the participants and multivariate logistic analyses of factors associated with dyslipidemia. With respect to BMI, WC, BP and FBG, the prevalence of high TC, LDL-C and TG were closely related with obesity (adjusted OR = 1.421, 1.143, 1.767, respectively), hypertension (adjusted OR = 1.431, 1.279, 1.600, respectively) and DM (adjusted OR = 1.570, 1.359, 1.814, respectively). The prevalence of low HDL-C was associated with obesity and DM (adjusted OR = 3.324, 2.673, 4.134, respectively), but not with hypertension. Only the prevalence of high TG was found to increase with WC (adjusted OR = 1.473, *P* < 0.001).

With regard to current smoking and alcohol consumption, we observed an association between cigarette smoking and the prevalence of low HDL-C and high TG (adjusted OR = 1.242, 1.096, 1.408, respectively). In addition, a significant relationship was detected between alcohol consumption and prevalence of high TC (adjusted OR = 1.691, *P* < 0.001). However, the prevalence of low HDL-C was lower among current drinkers (adjusted OR = 0.310, *P* < 0.001).

As for education, an inverse relationship was observed between the level of education and the prevalence of dyslipidemia, mainly for high TC. Prevalence was the highest among illiterate individuals (24.9%) and the lowest among middle school educated individuals (12.8%), and then increased among the group of high school educated or above (14.8%). We determined a significant association between the prevalence of high TC and marital status. Compared with married or widowed subjects, unmarried persons had greater odds of having high TC (*P* = 0.015). Family income was associated with increased odds of having high TC and low HDL-C (*P* < 0.05).

**Figure 1:**
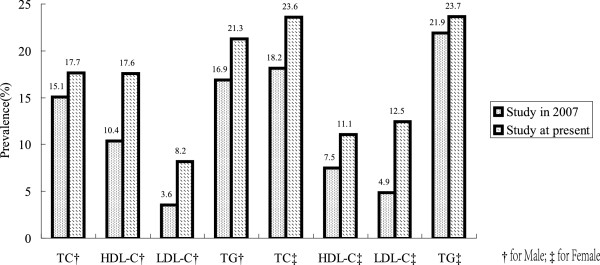
**Prevalence of high TC, low HDL-C, high LDL-C and high TG in hypertensive subjects between our current study and a study from 2007.**

We also conducted a subgroup analysis including 5919 hypertensive individuals (2892 men and 3027 women) and found significantly increased prevalence rates of dyslipidemia in Liaoning Province compared to the result of our past study including a total of 6,412 hypertensive individuals ≥35 years (2,805 men and 3,607 women) in 2007 [25]. From the result in Figure 1, we found that the prevalence in high TC, low HDL-C, high LDL-C and high TG had increased since these years in both men and women.

## Discussion

The prevalence of dyslipidemia is steadily rising in developing countries [26]. Dyslipidemia is one of the established conventional risk factors for CVD in the same category with other high-risk factors such as cigarette smoking, diabetes, and hypertension. Remarkably, these risk factors and their associated clinical outcomes can be largely controlled by adopting a healthy lifestyle and with proper medications. Therefore, management of dyslipidemia, and its associated risk factors is an important public health priority. The present study reports one of the largest population-based lipid studies ever conducted in China, in which current epidemiological characteristics of dyslipidemia in Liaoning Province were analyzed. In addition, the present study includes 85.3% of all permanent residents from the randomly selected 26 rural villages, so the study provides a very representative data applicable to Chinese population. Our study revealed an alarmingly high prevalence of dyslipidemia in China.

In our current study, the prevalence rates of abnormal lipid levels (including borderline dyslipidemia and dyslipidemia) were 47.8%, 13.8%, 25.7% and 30.7% for TC, HDL-C, LDL-C and TG, respectively, while the total prevalence rates of dyslipidemia were 16.4%, 13.8%, 7.6% and 17.3% for TC, HDL-C, LDL-C and TG, respectively. Therefore, 36.9% of China adults aged ≥35 years in this study had at least one type of dyslipidemia, which was higher than the Chinese National Nutrition and Health Survey conducted in 2002 (18.6%) [13]. Further, 64.4% had at least one type of abnormal lipid concentration, which was higher than the results from the National Health and Nutrition Examination Survey conducted in 2003–2006 (52.9%) [12], and the International Collaborative Study of Cardiovascular Disease in Asia conducted in 2000-2001 (53.6%) [14]. We conclude from our data that dyslipidemia is increasing at high rates in these populations, and dyslipidemia needs more urgent attention than ever before.

In hypertensive participants, the prevalence of high TC, low HDL-C, high LDL-C and high TG in Liaoning Province had increased significantly since 2007 in both men and women compared with our past study [25] as presented in Figure 1. Samples in the two studies conducted in 2007 and 2013 came from rural areas of Liaoning Province and had similar geographical and demographic characteristics. Therefore, our study provides quantitative measurements of what we believe are the true values of this increasing trend, based on our data analysis in this population within the same region. An increase in dyslipidemia may contribute to other complications of hypertension and higher mortality due to CVD. The changing lifestyle of these areas may account for the strikingly high prevalence of dyslipidemia. This study also revealed the possible risk factors for each kind of dyslipidemia by multivariate logistic regression analysis.

### Gender

The OR for the total dyslipidemia was greater in males than females, which is in accordance with most previous studies [13, 27]. However, one study indicated that the dyslipidemia is more prevalent in females [17]. Our study demonstrated that the prevalence of low HDL-C was higher in men and the prevalence of high TC and high LDL-C was higher in women, which was similar to the result of the Chinese national nutrition and health survey (CNHS) in 2002 [13].

### Age

It is worth noting that the prevalence of high TC, LDL-C and TG increased significantly with advancing age and thereafter began to decline, with the highest prevalence observed among age group 55–64, thus, all three values changed in parallel. However, the prevalence of low HDL-C declined at first and then increased along with advancing age, with the lowest prevalence being observed among the 55–64 age group. A similar trend of lipid distribution was found in the Trabzon lipid study, with highest prevalence of high TC, LDL-C and TG among the age group 60-69 [27].

### BMI, WC, BP and FBG

The relationship between dyslipidemia and the traditional factors including BMI, WC, BP, and FBG are presented in our study. The prevalence of high TC, LDL-C, TG, and low HDL-C were all closely related with BMI-defined overweight and obesity which was in agreement with those of previous studies [16, 28, 29]. Dyslipidemias are more prevalent in abdominal obesity than normal WC. But after adjusted for other factors, WC was an independent risk factor only for high TG. The possible reasons may be that participants in our study have less WC and the effect on lipid is corrected by co-existing factors. Hypertension increased the prevalence of high TC, LDL-C, and TG but not HDL-C which was in accordance with previous population-based study [16, 29]. In addition, similar with BMI, all kinds of dyslipidemia were positively correlated with DM in this study. The metabolic disturbances including disturbed glucose, overweight/obesity and abdominal fat distribution, dyslipidemia, and hypertension are often concurrent and strongly associated with subsequent development of CVD, with insulin resistance being the most important common mechanisms [30]. Our study finds that these metabolic disturbances showed increased prevalence in our study compared to previous studies, indicating the epidemic trend of CVD.

### Smoking and drinking

As an independent risk factor for CHD, smoking is a major health problem in China, with a prevalence of 57.1% in male and 16.6% in female participants in our resent study. We also determined an association between cigarette smoking and a prevalence of low HDL-C and high TG. In addition, a positive association was detected between alcohol consumption and high TC. However, the prevalence of low HDL-C was significantly lower among current drinkers, which was same as the result of the Trabzon lipid study [27].

### Education, marital status, family income

A general inverse relationship was observed between the level of education and dyslipidemia. Prevalence of dyslipidemia was the highest among illiterate individuals (42.3%) and declined along with increased education level with the lowest among middle school educated individuals (33.5%), and after that it increased among the group of high school educated or above (37.0%). This trend was shown specifically in TC and LDL-C which account mainly for the progress of CHD. The detailed association between marital status, family income and lipid level was also showed in Table 3, and high family income may lead to increased OR of having dyslipidemia.

There are some limitations in our present study. One limitation is that the dietary factors associated with dyslipidemia could not be analyzed due to lack of detailed dietary survey in our current study. Another limitation is that our design is cross-sectional, which could only reflect associations between dyslipidemia and risk factors.

In conclusion, our present study provides reliable and current epidemiological information regarding dyslipidemia among the adult population in Liaoning Providence, revealing very high prevalence of dyslipidemia, and calling for an urgent attention than ever before. We also examined various risk factors for dyslipidemia, some of which are independent risk factors for CVD. Our results are expected to be useful to develop appropriate community-based prevention strategies for these modifiable risk factors of dyslipidemia and reduce the overall CVD morbidity and mortality.
